# A Modular Approach for a Patient Unit for Extracorporeal Membrane Oxygenation Simulator

**DOI:** 10.3390/membranes11060424

**Published:** 2021-05-31

**Authors:** Yahya Alhomsi, Abdullah Alsalemi, Mohammad Noorizadeh, Faycal Bensaali, Nader Meskin, Ali Ait Hssain

**Affiliations:** 1 Department of Electrical Engineering, Qatar University, Doha P.O. Box. 2713, Qatar; aa1300250@qu.edu.qa (A.A.); m.noorizadeh@qu.edu.qa (M.N.); f.bensaali@qu.edu.qa (F.B.); nader.meskin@qu.edu.qa (N.M.); 2 Medical Intensive Care Unit, Hamad Medical Corporation, Doha P.O. Box 3050, Qatar; ahssain@hamad.qa

**Keywords:** simulation-based training (SBT), extracorporeal membrane oxygenation (ECMO), blood oxygenation, thermochromism, high-fidelity simulation

## Abstract

Despite many advancements in extracorporeal membrane oxygenation (ECMO), the procedure is still correlated with a high risk of patient complications. Simulation-based training provides the opportunity for ECMO staff to practice on real-life scenarios without exposing ECMO patients to medical errors while practicing. At Hamad Medical Corporation (HMC) in Qatar, there is a critical need of expert ECMO staff. Thus, a modular ECMO simulator is being developed to enhance the training process in a cost-effective manner. This ECMO simulator gives the instructor the ability to control the simulation modules and run common simulation scenarios through a tablet application. The core modules of the simulation system are placed in the patient unit. The unit is designed modularly such that more modules can be added throughout the simulation sessions to increase the realism of the simulation sessions. The new approach is to enclose the patient unit in a trolley, which is custom-designed and made to include all the components in a modular fashion. Each module is enclosed in a separate box and then mounted to the main blood simulation loop box using screws, quick connect/disconnect liquid fittings, and electrical plugs. This method allows fast upgrade and maintenance for each module separately as well as upgrading modules easily without modifying the trolley’s design. The prototype patient unit has been developed for portability, maintenance, and extensibility. After implementation and testing, the prototype has proven to successfully simulate the main visual and audio cues of the real emergency scenarios, while keeping costs to a minimum.

## 1. Introduction

**Figure 1:**
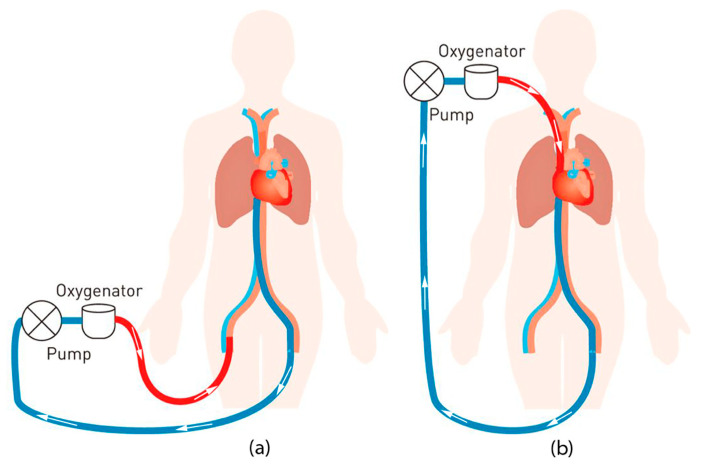
Illustration of the ECMO circuit. (**a**) Represents venous-arterial and (**b**) represents venous-venous setup. (Red is oxygenated blood while blue is deoxygenated blood).

Extracorporeal membrane oxygenation (ECMO) is an advanced procedure that can increase the recovery rate of patients with temporary respiratory and/or circulatory complications. The procedure is based on circulating the patient’s blood through an outside oxygenator by external tubes to oxygenate and extract carbon dioxide from the blood and pumping the blood back to the patient. Figure 1 shows the basic ECMO circuitry. 

Despite many advancements in the ECMO procedure, which lead to improved survival rates, the procedure is still correlated with a high number of patient-side health complications [1]. Such complications are caused by the necessity of circuiting the blood out of the patient body with the ECMO machine. Monitoring those complications requires an ECMO multidisciplinary team (i.e., physician, perfusionist, respiratory therapist, ECMO nurse, bedside nurse) as well as an ECMO nursing staff member to monitor the patient twenty-four hours a day. The trained nurse needs to watch over fifty variables such as flow, pressure, oxygenation levels, and temperature [2]. Furthermore, they need to develop swift responses to assess the situation quickly and address it confidently. Teamwork and communication are also important since most, if not all, ECMO-related complications require complicated procedures to be carried out on the spot; thus, ECMO staff should be able to effectively communicate and coordinate to increase the odds of patient survival. 

Offering medical staff an advanced training is based on two important factors. The first factor is theoretical understanding by attending lectures and classroom-based learning about the ECMO operation and how it works. The second factor is hands-on experience from real-life medical emergencies, dealing with patients, and applying what was learned in the classrooms. The latter comes in conflict with the duty of providing the highest quality care possible to ensure patient’s safety, especially in the intensive care units and especially when medical equipment are used for education purposes [3].

To address the needs of both patient care and staff training, medical educators have deployed simulation-based training (SBT) techniques to offer an alternative for the actual medical emergencies by implementing high-realism simulation sessions [3]. Different studies show that SBT improves communication, behavior, and technical skills on mannequins. Such improvements can potentially translate into better patient care [3,4,5]. SBT allows the trainees to safely understand the ECMO procedure, the patient interactions with the ECMO machine, enhance the team cooperation and execution during emergencies, and develop the skills of an ECMO specialist [6,7]. However, ECMO SBT practices include basic mannequin modifications, remotely controlled mannequins, and full-patient simulators [4,5,6,7]. These practices have remarkable disadvantages such as utilization of high-cost, one-time-use medical equipment, manual modification of the circuit, and the inability to generate the effect of blood oxygenation color change. Therefore, a re-invention of the conventional medical simulator architecture is inevitable [8].

In this paper, in order to overcome the aforementioned obstacles, a “revolutionary“ simulator design model is presented with two main concepts: modularity and physical fidelity. The former term is defined as the decomposition of a system into small subsystems (or modules) independently developed yet functional together as a whole via standardized interfaces [9,10]. Utilizing modularity can result in lower overall life-cycle cost, in addition to being extensible. Due to their independent design, modules can be further developed, as needed, or even replaced by other modules to boost the performance of the system. The second cornerstone concept is the physical fidelity. It is defined as the degree of apparent resemblance of a phenomenon visually, audibly, and haptically [10]. This implies that a successful simulation mimics the visual/audio cues of a given phenomenon, not necessarily reproducing it physiologically [11]. Modular components must satisfy physical fidelity to achieve high-fidelity simulation. It starts by listing the objectives and design constraints of the simulation system, then transforming the objectives into simulation modules. For each simulation module, a mechanism is developed to simulate a certain physical cue. Then a real-time, wireless communications scheme is put into place to allow for all the simulation modules to coordinate with each other. The design is then implemented and evaluated on the basis of the simulation objects and the high realism criteria. 

Building upon the aforementioned framework, the prototype ECMO simulator is developed for the use of patient management training at Hamad Medical Corporation (HMC), the main public healthcare provider in Qatar. The system revolves around the use of thermochromic ink to simulate blood oxygenation, deoxygenation, and recirculation. Thermochromic ink is a special substance that changes color with temperature variation. This is where the physical fidelity comes into play. Thermochromic ink is used as an alternative to real blood due to the high-cost and disadvantages of using real blood. The purpose of using real blood is to generate the visual cue of color change between red and dark red, where red is oxygenated blood and dark red is deoxygenated blood. Thermochromic ink can be used to provide the same visual cue while avoiding the real blood hurdles and its high cost. On top of the thermochromic loop, ECMO complications are simulated via several modules. The line chattering module simulates high negative pressure at the drainage tube using a linear motion device. Hypovolemia is simulated using a pump that pushes thermochromic fluid from a reservoir tank. Pump air noise is simulated using a speaker placed inside a mock oxygenator unit, and so on. ECMO instructors control the simulator through a special tablet application that stores all ECMO parameters in a real-time cloud. These parameters are correspondingly displayed on an emulated console of the ECMO machine. 

The core unit, i.e., the patient unit, is designed using the thermochromic ink loop that enables the simulation system to be modular. The patient unit is a multi-pane holder unit that can facilitate the installation of different modules to effectively increase the physical fidelity of the simulator. The modularity is achieved by two main features. The first feature is providing an easy mounting mechanism to the main patient unit case. This allows new modules to get power from the main unit and connect easily to the main thermochromic loop. The second feature is the ability to connect to the main control board. This enables the instructor application to connect and control the new added module with the need to reprogram or design a whole new control board for just adding a new simulation module. Both discussed features allow for rapid, easy, and low-cost maintenance and upgrade. 

The remaining sections of this article are structured as follows. Section 2 reviews the related contributions and the unique features about the simulator based on the patient unit. Section 3 briefly describes the different components of the proposed simulator and provides detailed information about the patient unit design. Section 4 reports and discusses the obtained results of the implemented patient unit and its effectiveness. Section 5 concludes the article.

## 2. Related Work

In complex medical procedures, such as the ECMO procedure, hands-on training is a key factor for the medical staff to understand the flow of the procedure and interaction between the ECMO machine and the patient. In addition, the medical staff is trained on emergency scenarios using the simulator to gain the fast interventions and actions required as a team in order to treat life-threatening complications within a small time window [12]. Simulation technologies and methodologies differ with different training centers since, to the authors best knowledge, there is no comprehensive standard simulation procedure for ECMO. However, there are guidelines set by the Extracorporeal Membrane Life Support Organization (ELSO) [13].

### 2.1. Common Simulation Methods

ECMO SBT is still in its early development stages [8]. Only recently has the demand for technologies to specifically address ECMO increased, especially after the surge of the recent coronavirus pandemic (COVID-19) [14,15]. Hence, instructors had to use available equipment and resources to create relatively immersive ECMO emergency scenarios, leading to a common simulation scheme throughout the literature [16]. 

The first ECMO SBT was reported by Anderson in 2006 [17,18] where the instructors at Lucile Packard Children’s Hospital simulated a neonatal intensive care unit by using functional medical equipment including an ECMO circuit and ECMO code carts. The ECMO circuit consisted of a roller pump head, an oxygenator, a heat exchanger, and a bladder. The circuit components were interconnecting by ¼” Tygon tubes and connected to a neonatal low-fidelity mannequin via 12 Fr arterial and 14 Fr venous cannula inserted into the mannequin right neck. The simulated vitals were displayed using a bedside 4-monitor and pulse oximeter, both remotely by the instructors. The pressure in the circuit was set manually by adjusting the occlusion of the roller pump. The remotely controlled patient parameters were used to create theoretical emergencies like cardiac tamponade and acute hypoxemia. 

The ECMO simulation method preformed at Lucile Packard Children’s Hospital was well received by participants, and Anderson reported a significant increase in the learners’ technical and behavioral skills [17,19]. A trend of other hospitals reporting the use of similar methodology appeared in the literature, and the common aspects consisted of (1) the use of functional medical devices including an ECMO circuit; (2) modifying/drilling mannequins to connect the circuit internally; (3) use of remotely controlled patient monitors to display simulated vitals; and (4) concealed adding and removal of liquid and air to change circuit behavior causing pre-set alarms to activate. ECMO circuit equipment has advanced over time, however, the incumbent simulation method has remained applicable. In 2012, Brazzi reported using a SimMan mannequin alongside a PLS system (MAQUET), which uses a centrifugal pump and a modern polymethylpentene (PMP) oxygenator [7]. The use of the modern and popular ECMO CARDIOHELP system and HLS oxygenator set was reported by Ng in 2016 in simulation centers in Hong Kong. 

### 2.2. Technological Enhancement

In recent years, circuit enhancements were used to automate some of the challenging tasks of the common method. In 2012, the Orpheus perfusion simulator was first incorporated into a simulated ECMO circuit [20], and the mannequin was modified to pass the PVC tubes coming from the ECMO to the Orpheus without being noticed by the trainees [20]. The simulator is connected in series between the arterial and venous tubes, and it hydraulically replicates the native heart, arterial system, and venous system’s capacitance using the Frank–Starling model. The Orpheus is incapable of creating venous pressure, a reading needed in ECMO circuitry; hence, similar to the classical method, a bladder was incorporated to the venous line to create changes in the venous pressure. Using this setup, the simulated ECMO circuit is now capable of creating age-realistic circuit parameters (pressure and flow rate), and thus it easier for instructors to simulate high-risk scenarios such as tamponade, oxygenator failure, kinked tubes, and air entertainment. Additionally, in [21], the NIJMEGEN ECMO Simulator is a relatively advanced system, where it can simulate typical parameters that can be wirelessly controlled. The packaging is also considered portable. Moreover, research efforts by Puślecki et al. [22,23] propose a low-cost ECMO mannequin that includes a controllable hydraulic system, allowing for pressure manipulation. It also supports cannulation simulation.

There is a number of commercially available ECMO simulators that work as add-ons connected in series to the ECMO common simulation circuit. Examples are EigenFlow and Parallel Simulator. Both simulators can manipulate the circuit pressure, and they include a remotely controlled screen that provides parameters not available in standard bedside monitors, such as venous and arterial line oxygen saturation [24,25]. Another case study is ECMO Patient Simulator (EPS) by Biomed Simulation Inc (San Diego, CA, United States). [26]. Used for surgical cannulation and connection to the ECMO machine, the simulator uses artificial blood to simulate different related scenarios.

### 2.3. Drawbacks 

Despite the many efforts, there are multiple issues with contemporary simulation methodology. First, they are inherently expensive as they generally rely on using disposable medical equipment (e.g., oxygenators, heat exchanger, pumps), which need to be replaced occasionally due to the damage the additive coloring causes. Furthermore, circuit alarms need to be manually triggered, and there is no standard calibration to obtain pressure values. 

Expensive add-ons to the circuit enhance the process by giving the instructors an easier option to change circuit pressure and display blood parameters. However, blood parameters are usually displayed on an additional screen, which feels like a gimmick to systems with available blood parameter displays. The add-ons do not address the main issues with the simulation method (e.g., continuously having to replace circuit components). In addition, the simulation system does not visually simulate the primary function of ECMO, oxygenation. Food coloring keeps the circuit mono-colored, which makes the process less realistic. Table 1 compares recent related work.

In this work, in order to advance upon the reviewed literature, a novel ECMO SBT solution is presented. Specifically, we tackle the development of the patient unit, which is responsible for blood simulation, among other simulation modules needed in ECMO simulation and missing from the literature. 

The authors of the paper have published work related to the novel simulator [10,27,28,29]; however, this work focuses on the contributions enabled by the design and implementation of the patient unit, which has not been explored before.

## 3. Materials and Methods

### 3.1. Simulator Overview

**Figure 2:**
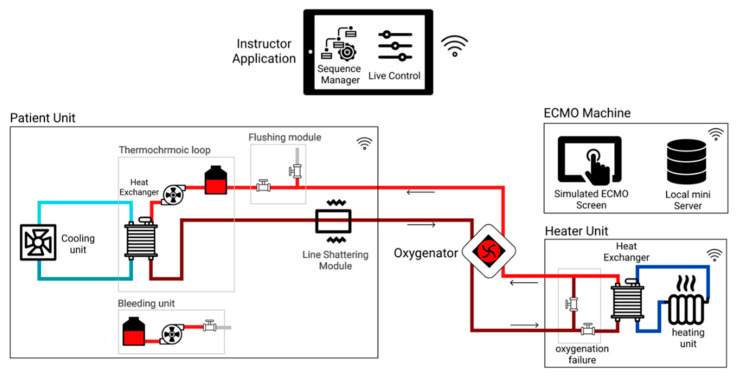
Overview of the ECMO simulation system.

To recreate the ECMO circuit, there is no need to actively try to achieve realistic operation conditions (e.g., pressure, flow rate, temperature); instead, the focus can be on recreating the observed appearance. Hence, we emphasize on creating the visual and audio effects of the ECMO procedure and circuit functionality. To implement such a simulator, there are four main units that work together to create the necessary visual and audio effects; all units are controlled and monitored by the instructor through the instructor tablet application [30]. The proposed simulator can be used as an independent simulator that does not depend on other medical devices or simulation systems. The simulator can run basic ECMO training programs or can be easily upgraded to run advanced training programs by adding modules that will mimic the visual or audio cues of an emergency scenario, such as line shattering, which is discussed in Section 3.3.3. Figure 2 presents an overall view of the system, the flow of the circuit, and where each component is placed in the simulation system.

#### 3.1.1. Patient Unit 

The patient unit is where most of the simulation modules are placed along with the thermochromic loop [28], which makes the patient unit the core unit of the simulator. The function of the patient unit is to start and maintain the thermochromic loop and allow for the addition of extra simulation modules in a modular fashion. This allows to expand the simulator functionally and the number of scenarios that can be simulated. The patient unit main control board will be able to supply the simulation module with power and control signals. This eliminates the need to have a microcontroller, communication module, power supply, and use a medical grade device to mimic the visual and audio cue of a real scenario. Thus, the cost of the modules is dropped significantly compared to other medical grade modules used in other simulation systems that are controlled independently and cannot sync together to form a complete simulation scenario. Additional simulation modules are usually placed within the patient unit due to the location of the unit. The unit is placed underneath the bed or beside the mannequin bed. This allows to add more modules to simulate patient-related visual and audio cues, where most of the emergency scenarios happen.

#### 3.1.2. ECMO Unit

The ECMO unit is a replica of the original ECMO machine that drives the oxygenator and displays all the values related to the patient and the control pump. To replicate such a machine, the team has created a casing containing a single-board computer that runs a software with the same original user interface to allow the trainee to go around the software and try out different options, similar to the original ECMO machine. The instructor is also able to remotely control the displayed values on the screen and display different alarms to test the trainee. 

#### 3.1.3. Heater Unit

The heater unit completes the cycle of the thermochromic loop with the patient unit to achieve color change [31]. The heater unit is where the thermochromic loop gets heated to turn its color from dark red to light red, representing the change of the deoxygenated blood to oxygenated blood that is coming through the mock oxygenator mounted on the ECMO machine. 

#### 3.1.4. Oxygenator 

Replicating the oxygenator of the ECMO machine is also an important factor for making a cost-effective and a modular simulation system, since the original oxygenator is quite expansive for running SBT. The replicated oxygenator is 3D-modeled to match the design and the feeling of the original design. In addition, the mock oxygenator is remodeled internally to redirect the thermochromic loop to complete the cycle with the heat unit and go back to the patient unit. A matrix of addressable LED lights and small speakers are placed inside the oxygenator to simulate blood clots and air bubbles. Both cues can be controlled by the instructor app.

### 3.2. Patient Unit Design

The patient unit is the core unit of the ECMO simulation system. The first factor that makes the patient unit as the core unit of the system is that it contains all essential components that are responsible for most of the simulation scenarios. The unit contains the main control board, thermochroic ink tanks and pump, heater/cooler module, main power supply, and different simulation modules. Another factor is that most of the physical and visual cues take place at the patient side. Thus, most of the simulation modules are placed at the patient unit, which are all controlled by one controller and powered by the same power supply. 

Building the patient unit as a modular unit plays an imperative role in making the whole simulator as a modular system. In order to consider a unit as a modular unit, the instructor should be able to add, remove, upgrade, and perform maintenance on the simulation modules separately without the need of reprograming or redesigning the whole unit. Such criteria require the patient unit to have the following:

1. An enclosure that allows for other modules to attach to the main unit case.
2. Quick connect and disconnect tube connection fittings between modules.
3. Wi-Fi-enabled controller with extra control connections for adding new modules.
4. Independent and portable unit.
5. Fast and easy initial setup.

In order to meet the above criteria, we have followed the pragmatic approach of physical fidelity married with modularity to develop features that will result in a modular patient unit, and ultimately a modular ECMO simulator.

#### 3.2.1. Cooling System

The thermochromic loop requires a constant supply of cold water in order to cool down the thermochromic ink and turn the color from light red to dark red. Thus, an external water chiller should be connected to the patient unit, where the thermochromic ink loop is based. The need for external water makes the patient unit dependent on an external water chiller. For this reason, the team has decided to develop a compact water cooler unit that will be included in the patient unit [31]. 

**Figure 3:**
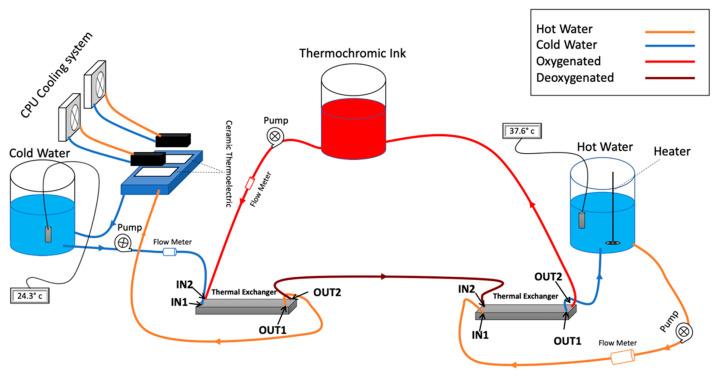
Heater/cooler block diagram [31].

As shown in Figure 3, the thermal exchanger has four ports: IN1, IN2, OUT1, and OUT2. Accordingly, the blood is delivered to IN1 and goes out from OUT1. Meanwhile, the cooling unit affects the blood entering IN2, and then it goes out from OUT2. Indeed, by placing two thermoelectric modules on the aluminum water/coolant cooling block, the temperature of the water/coolant inside this block will decrease. It will also circulate, by a controllable pump, between the cooling tank and the thermal exchanger unit. Moreover, two CPU cooling modules decrease the temperature of the heating side of the thermoelectric module. The unit components have been selected from the same manufacturer to ensure compatibility and fitting. Moreover, due to the considerable effect of flowrate on the cooling performance, several flowrate meters have been implemented in the circuit after each pump, and it is observed that by increasing the flowrate, the cooling effect dramatically decreases for the cooling unit. Hence, the flowrate is a significant key factor in this prototype, and by finding the trade-off between the flowrate and appropriate temperature, the overall performance can be optimized.

#### 3.2.2. Portability and Connections 

**Figure 4:**
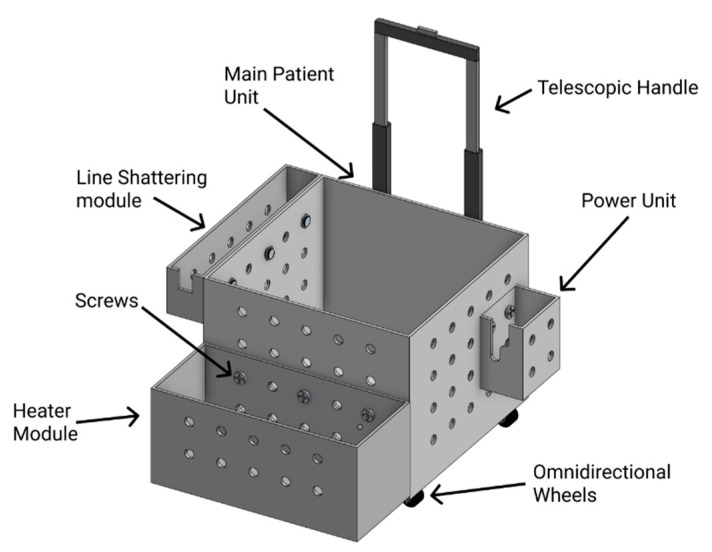
3D model of the patient unit modular trolley.

The approach is to enclose the patient unit in a trolley made of clear acrylic. The trolley is custom-designed and made to include all the modules in a modular method. Each module is enclosed in a separate acyclic box and then mounted to the main thermochromic ink loop box using screws, quick connect/disconnect liquid fittings, and electrical plugs. This method allows fast upgrade or maintenance for each module separately and allows to add future modules easily to the exiting patient unit without redesigning the patient unit trolley again. Additional wheel casters can be added to the additional module boxes to make it easier to carry around. Figure 4 demonstrates how each module box is attached to the main patient unit using screws to allow for simple expandability.

In this design concept, the number of connections needed for initial setup is minimized, where all connections are made inside the module boxes and only having minimal external connections to connected with other modules or other units.

#### 3.2.3. Automatic Fluid Flushing

The upgraded patient unit has a system of valves that can be controlled to run a mode called “flushing”. The flushing mode is responsible for automatic flushing of all the tubes and connectors of the thermochromic ink using water to avoid staining of the thermochromic ink powder in the liquid system. This staining effect can reduce the efficacy of the next simulation run and cause pipe clogs. This operation prevents the need to change the tubes of the system in each session, making the thermochromic ink the only element of the system that needs to be replaced in each session, compared to the high-cost animal blood, medical grade oxygenator, and other equipment that needs to be replaced each couple of sessions during other simulation systems. The flushing mode is started using the instructor application with few instructions to follow. The flushing was previously done manually where the whole liquid system was dissembled and cleaned invalidly. 

**Figure 5:**
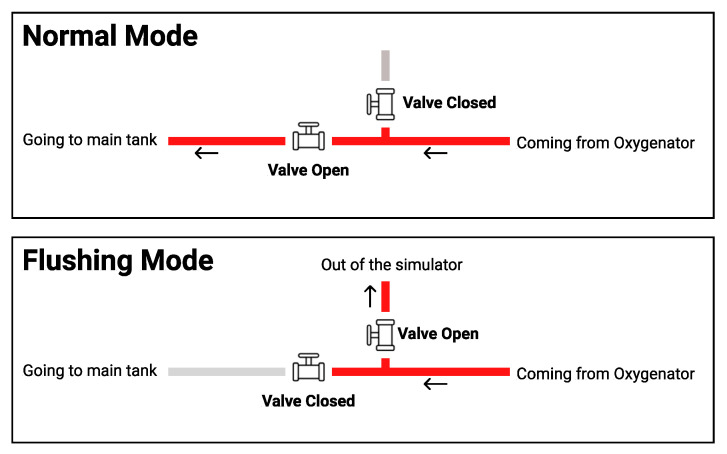
Path of the thermochromic loop in normal operation and in flushing mode.

As shown in Figure 5, in the normal operation, valve A stays closed, and valve B stays open to allow the thermochromic ink to go back to the main tank (tank A) and get pumped again to the loop through pump A. In the flushing mode, valve A becomes closed and valve B becomes open to force the thermochromic ink to be drained out of the system instead of going back to tank A.

#### 3.2.4. Main Control Board

To be able to control the thermochromic loop and other simulation modules within the patient unit, a control board is designed to pass the orders from the instructor tablet and send it to each module through a central local database. The control board is mainly responsible for reading the control values from the local database via the instructor application. The main control board should have the following specifications in order to control and allow for any additional modules that can be added later on after the initial implementation:

1. Power regulation circuit to power the simulation modules;
2. 50 MHz or faster microcontroller to analyze the database and send them to the modules in real time; and
3. Wi-Fi module to connect to the local database.

**Figure 6:**
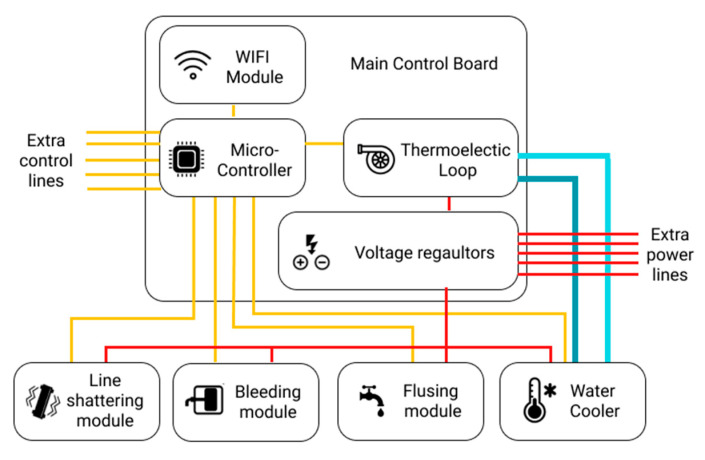
Block diagram of the main control board and module integration.

With these specifications, the main control board will be able to integrate with other modules. Figure 6 shows how these modules are integrated with the main control board. 

### 3.3. Patient Unit Simulation Modules 

The patient unit is responsible for powering and controlling different simulation modules that are placed at the patient side. These simulation modules have different functionalities and structures based on the visual and/or audio cues that are simulated. 

#### 3.3.1. Thermochromic Loop 

As mentioned in the previous sections, the main functionality of the ECMO machine is to oxygenate the blood that comes from the patient as deoxygenated and then return it to the patient [32]. During this process, the color of the blood changes between dark red and light red consistently. Thus, the most important visual cue of the ECMO operation is the color change of the blood in the tubes between the patient and the ECMO machine. 

**Figure 7:**
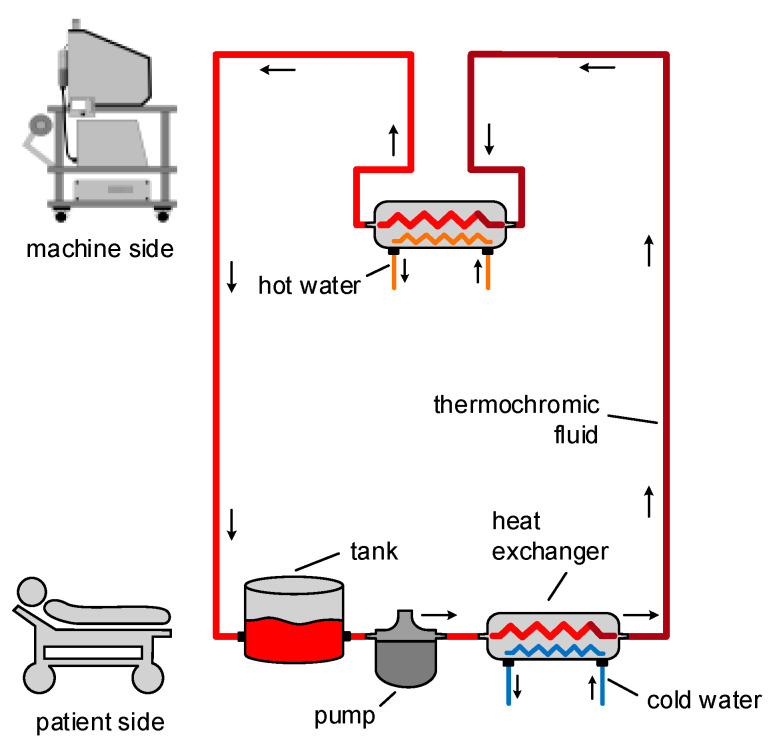
Thermochromic loop structure.

To simulate such a color change, the team has developed a system that utilizes thermochromic ink, which resulted in a low cost, efficient, and durable color change simulator. The thermochromic loop consists of two main units. As Figure 7 shows, the first unit is at the patient side where the ink gets colder to change the color from light red to dark red. The second unit is at the machine side, where the ink gets warmer to change the color from dark red to light red. This configuration allows to have control over the color change throughout the loop, which can be used to replicate real-life scenarios. Although the thermochromic ink has an effective time frame of 10 h, which after that it will start to lose its effectiveness to change the colors, the cost of replacing the ink is around 80 USD for each session. Compared to medical grade disposable equipment used in normal simulation sessions, this price is almost one-tenth of the cost. Moreover, the use of a medically safe ink such as the thermochromic ink can effectively replace the other possible options such as using a real ECMO machine that can be damaged during the simulation or even using possibly contaminated animal blood. 

#### 3.3.2. Patient Bleeding (Hypovolemia)

**Figure 8:**
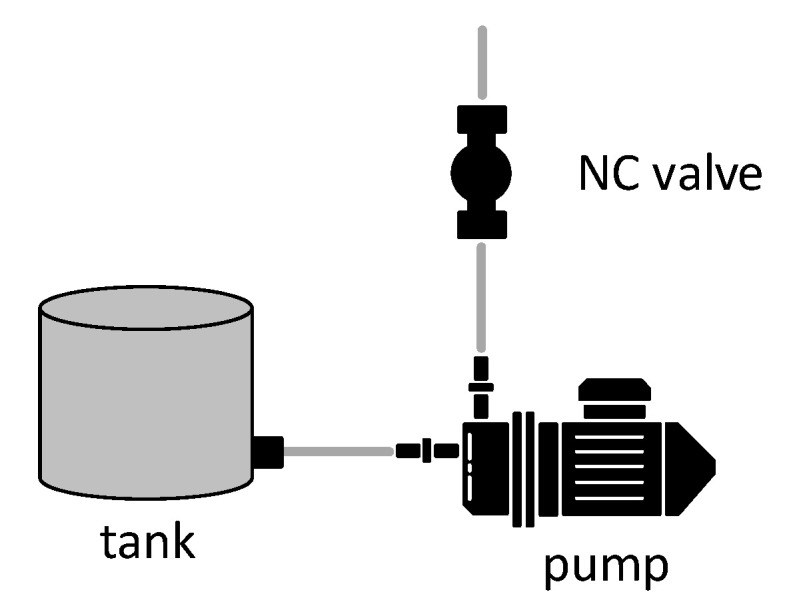
Patient bleeding module.

Hypovolemia is the loss of fluid in the circuit, which results in patient bleeding through the chest bladder in addition to the chattering of the tubes between the patient and ECMO machine. As shown in Figure 8, to simulate the patient bleeding, a pump is controlled to pump colored water from a separate tank (than the main loop tank) to a bleeding bag that is usually connected to the bladder of the patient. The pump is connected to the main patient unit board to control the flow speed of the pump based on the simulated scenario. 

#### 3.3.3. Line Chattering 

**Figure 9:**
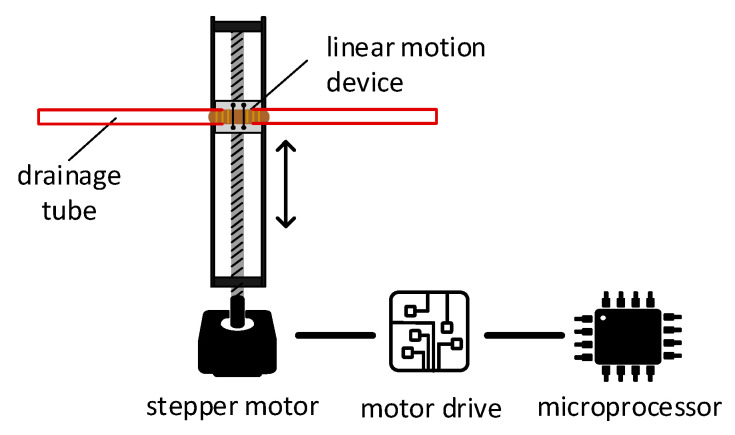
Line chattering module.

When the drainage negative (suction) pressure is too negative, the drainage tube starts vibrating. To create the vibration movement, a linear motion mechanism is used. The mechanism illustrated in Figure 9 converts the rotational movement of the stepper motor into a linear motion. The horizontal motion of the device corresponds to the direction of rotation of the electric motor (i.e., rotating clockwise will result in rightward, and vice-versa). As illustrated in Figure 9, this principle of operation can be employed to create rapid right and left movements, simulating vibration. The drainage tube of the ECMO circuit will be clipped into the moving head of the linear motion device, which should be small enough to be concealed within the patient bed. A microcontroller will control a stepper motor to rotate in specific angles to recreate line chattering. 

The mechanism used in the linear motion device is based on a belt-driven mechanism. This mechanism is based on rotating a pulley that can drag a belt connected to a linear motion slider. When the pulley is rotated and the belt is dragged, the linear slider will move linearly with the belt, and thus a linear motion is created. 

To implement the line chattering design, support parts are designed and 3D-printed to mount the parts together. The stepper motor’s torque is enough to vibrate a tube full of liquid, and the microcontroller accurately controls the rhythm of the vibration, allowing further customization. The module can be hidden under a bed sheet, where the top cover will prevent the bed sheet from interpreting the movement of the tube. To calibrate the module properly, an end-stop switch is used to calibrate and reinitiate the position of the tube during simulation setup. 

### 3.4. Instructor Application

The instructor application is the main control panel for the simulator. The instructor can install the application on their tablet devices, connect to the simulator local Wi-Fi network, and start controlling the simulator modules, values that appear on the ECMO machine screen, and run emergency scenarios [33]. The scenarios can be customized and predefined before the training sessions to simulate a full real-life scenario that can execute a series of visual and audio cues, allowing the instructor to focus on the learner actions and behavior during the simulation sessions instead of being occupied by hastily controlling the modules and adjusting values. 

**Figure 10:**
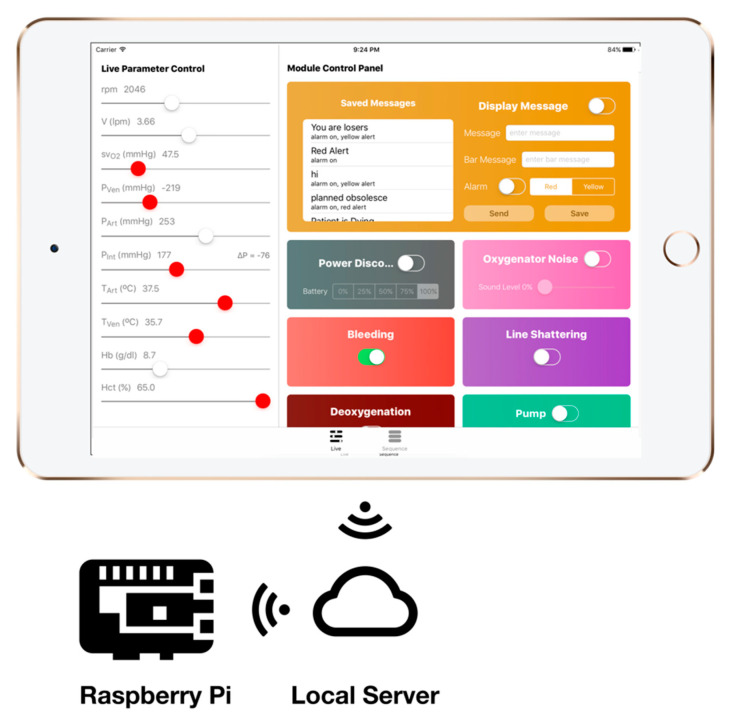
The instructor application.

The instructor application pushes parameter updates to a local database that is based on single computer board inside the ECMO machine case [34]. Other modules and units connect to the same database and execute based on the change of values on the database. Figure 10 shows the instructor application.

## 4. Results

Modules and mechanisms discussed in Section 3.2 were tested in HMC by five physicians, five perfusionists, eight nurses, and one respiratory therapist who are part of the ECMO education team at HMC. The study of measuring the effectiveness of the implemented simulation modules was conducted with a group of ECMO nurses and specialists at HMC. The results of the study scored on average 4.7 out of 5 on the responsiveness of the instructor app with the simulation modules and 4.8 out of 5 on average regarding the live control panel. Both scores indicate how simulation modules can be successfully controlled precisely to generate effective physical cues and the easiness of controlling them through the instructor apps. The next step is to take the simulation system to a product level and join all of these modules into a modular unit (patient unit) that can improve the effectiveness in addition to reducing the future development cost.

This section discusses the implementation of the patient unit prototype and its modules. Section 4.1 discusses the implementation of the patient unit done in two phases based on the constrains mentioned in Section 3.2. Section 4.2 illustrates the implementation of the modules placed in the patient unit. 

### 4.1. Patient Unit Implementation

This section discusses the implementation of the patient unit based on the constrains mentioned in Section 3.2.

#### 4.1.1. Phase 1: Modular Control Board 

**Figure 11:**
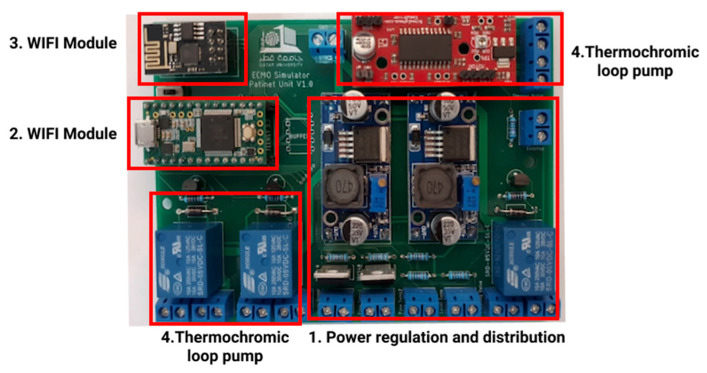
Patient unit main board.

In this phase, the main control board was designed to provide the modules with two main functionalities: power and connection to the local database. As shown in Figure 11, the main control board consists of the following parts:

1. Power regulation and distribution: this part provides different voltages and power options for the modules. This allows for different modules to get the needed power without the need of adding a new power module.
2. Microcontroller: the Teensy 3.2 Microcontroller reads the data from the database and then sends orders to the corresponding module and updates the database on the basis of the values given by the modules. In addition, Teensy 3.2 is also responsible for operating the thermochromic loop and making sure there is no clogs in the tubes through two flow sensors that are constantly checking the flow.
3. Wi-Fi module: the Wi-Fi module is the connection bridge between Teensy 3.2 and the local database to get data.
4. Thermochromic ink control module: this part controls the pump and reads the flowrate from the flow sensors.

#### 4.1.2. Phase 2: Modular Casing 

**Figure 12:**
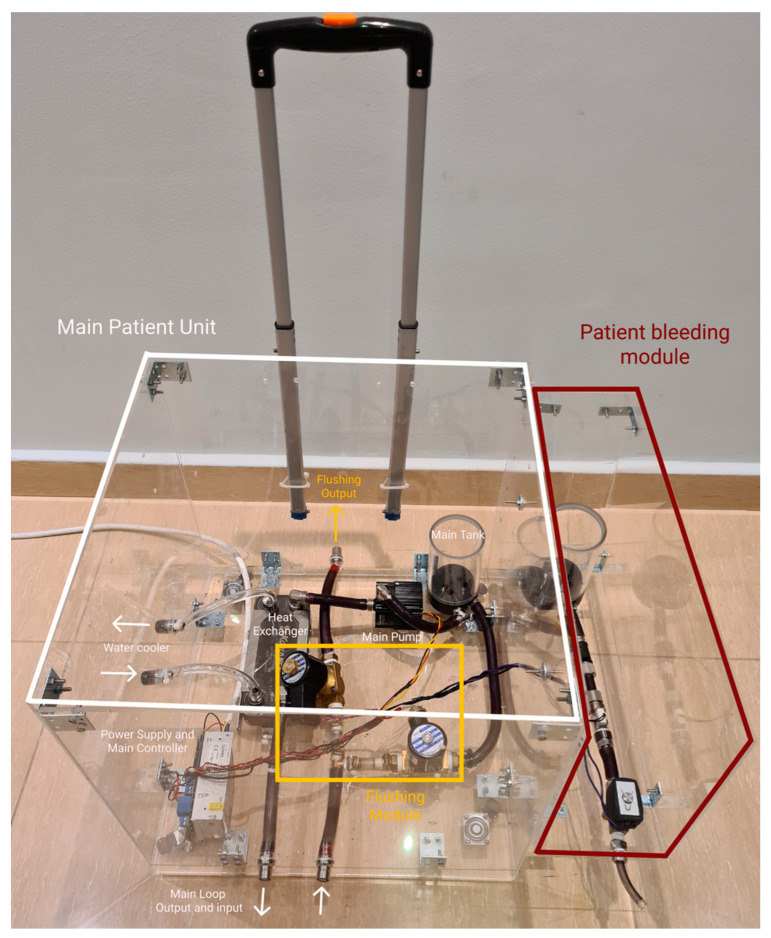
Patient unit modular casing implementation with patient bleeding module.

The implementation of the case was done by using clear acrylic sheets that were 8 mm thick. The sheets were cut using a laser cutter and assembled using M4 screws. Figure 12 shows the main patient unit case, with the patient bleeding module attached to it using screws, while sharing the power and the control lines from the main unit as discussed in previous sections. 

### 4.2. Simulation Modules Implementation

This section discusses the implementation of the simulation modules placed in the patient unit. 

#### 4.2.1. Automatic Fluid Flushing

**Figure 13:**
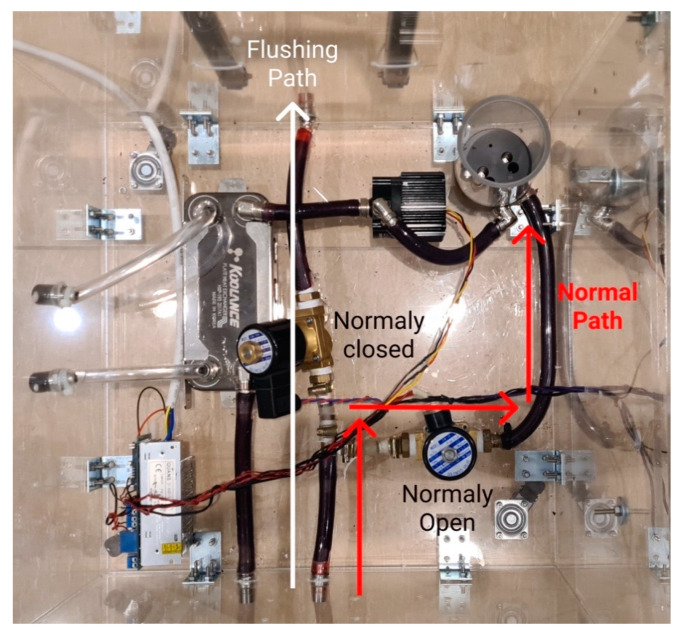
Implemented automatic fluid flushing module.

Per the aforementioned design in Figure 5, two valves must be placed between the thermochromic ink loop between the oxygenator and the main tank in a T-shape arrangement. The in-line valve is a normal open valve, which is always open unless power is supplied. The valve that leads to the outside of the circuit is a normal closed valve, which is always closed unless power is supplied. In normal operation, the two valves are off. Thus, the circuit is connected to the main thermochromic ink and continues circulating. In flushing mode, the circuit is discounted through powering both valves, which will redirect the path of the thermochroic ink to the outside of the circuit. This will empty the tank and tubes of circuit and make sure the ink will not precipitate in tubes and prevent future clotting. Figure 13 shows the implementation of the flushing module in the patient unit.

#### 4.2.2. Thermochromic loop

**Figure 14:**
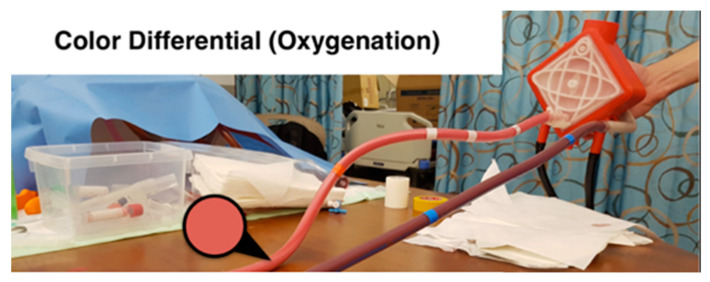
Thermochromic loop being tested at HMC.

Following the implementation of the thermochromic loop with the design discussed in Section 3.3.1 and the tests conducted at HMC, the thermochromic loop gave a clear color change between the inlet and the outlet of the oxygenator. Figure 14 shows the color change between the main two thermochromic lines when tested at HMC. 

#### 4.2.3. Patient Bleeding

**Figure 15:**
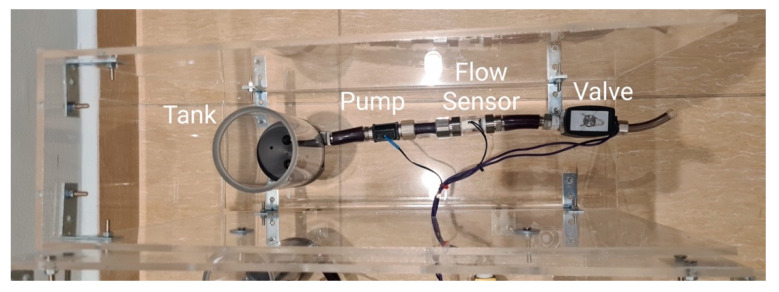
Patient bleeding module.

The patient bleeding module is implemented by using a separate pump to pump the colored blood from a separate tank to avoid interrupting the thermochromic ink. The outlet tube of the module is connected to a bleeding bag that is usually placed at the patient bed, which the nurses can easily see and, thus, take action if blood is found in the bag. Figure 15 shows the implemented module. 

#### 4.2.4. Line Chattering

**Figure 16:**
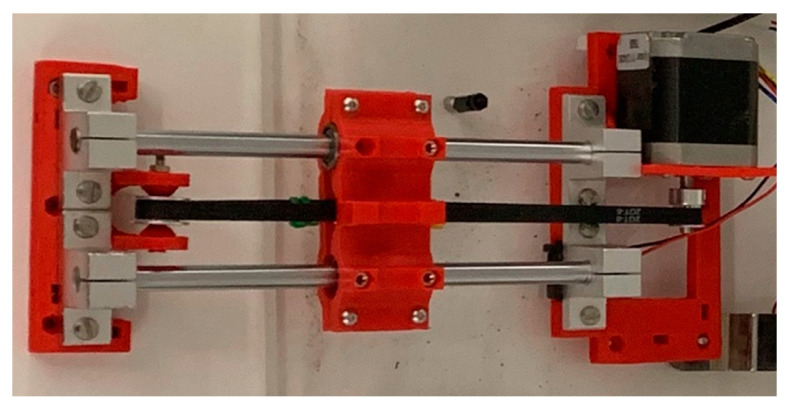
Line chattering module.

To implement line chattering design, support parts are designed and 3D-printed to mount the parts together. The stepper motor’s toque is enough to vibrate a tube full of liquid, and the microcontroller accurately controls the rhythm of the vibration, allowing further customization. The module can be hidden under a bed sheet, where the top cover will prevent the bed sheet from interpreting the movement of the tube. To calibrate the module properly, an end-stop switch is used to calibrate and reinitiate the position of the tube during simulation setup. Figure 16 shows the implemented setup of line chattering connected to a tubing.

#### 4.2.5. Cooling Unit

**Figure 17:**
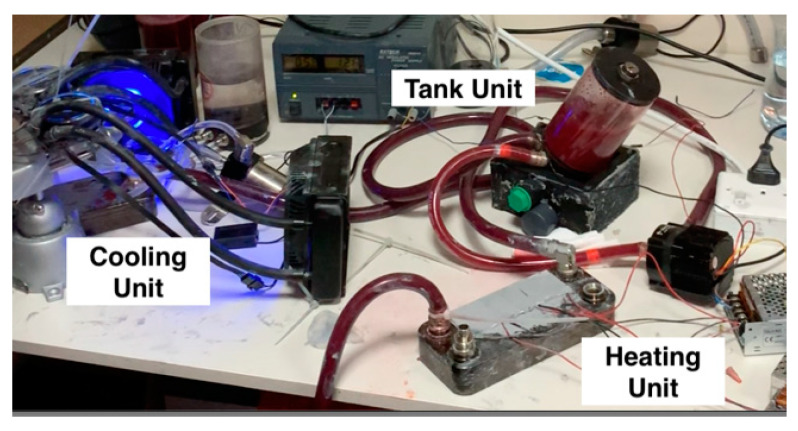
Current prototype of thermochromic heater-cooler system.

Implementing the cooling unit is done through utilizing the thermoelectric modules, as explained in Section 3.2.1. On the left side of Figure 17, the cooling unit is connected to the heat exchanger that is part of the main thermochromic loop. After the thermochromic ink passes through this heat exchanger, the color will change from light red to dark red, indicating the oxygenation of the blood. 

## 5. Discussion

High-fidelity training activities aim to achieve learner suspension of disbelief to prevent negative learning, the foundation of successful SBT. High-realism systems and configurations, though, are costly and require a considerable amount of budgetary investment. This is exacerbated by ECMO SBT techniques currently in usage. In addition to simulation services, ECMO centers offering SBT also rely on a functioning ECMO environment alongside expensive consumables.

The advantages of deploying our proposed ECMO training framework include lower maintenance and maintenance costs, as well as increased customizability and expandability. Overall, this breakthrough would significantly support the new ECMO SBT programs in order to further enhance the learning of their members. Current shortcomings of the training scheme involve inadequate integration with the ICU, which can impact the expected suspense of disbelief. It is our upcoming job strategy to overcome these constraints.

Owing to the severe and far-reaching effects of the COVID-19 pandemic, ECMO centers have registered an outstanding number of patients. In certain centers, the ICU achieves the full ability needed by novel and rapid methods for dealing with the influx of patients. From a first-hand view, in HMC’s medical ICU, overloading of personnel and ECMO machines allowed additional ECMO machines and facilities to be used for patients. The machines may be of a new brand, foreign to the workers, and in certain situations involve prior theoretical and practical experience. This was carried out in the context of a hypothesis combined with rapid simulation crash courses in order to familiarize the ECMO team with the subtle yet critical complexities of the different ECMO system models. 

From a technology point of view, designing a patient unit for an actual ECMO machine is very useful, in terms of preserving an additional device for specific needs and creating a simple on-the-go testing platform when the time is extremely important. In addition, the use of software is becoming increasingly crucial, particularly where social distancing has to be respected. As an example, the usage of the tutor tablet program helps the learner orchestrate and evaluate in real time, thus helping the teacher to retain a healthy physical distance. Overall, SBT tends to play an instrumental role in the training of employees for crisis management.

In terms of current limitations, and based on the feedback from the ECMO education team at HMC, the proposed patient unit is considered bulky in terms of size and weight, slightly unintuitive to initially setup, requires ink replacement every 10 h, and can be prone to liquid leaking if not sealed properly. The aforementioned limitations are being addressed and will be overcome in upcoming prototypes. The team is currently exploring the possibility of spraying the inner surface of the tubes with waterproof coating that will stop the ink particles from precipitating, thus increasing the lifetime of the thermochemical ink within the main loop.

The patient unit is to be integrated with a simulator, instructor application, and assessment application interconnected through the CouchDB communication system. Finally, the system is to be tested through a comprehensive study at HMC to evaluate the overall educational efficacy of the proposed solution.

## 6. Conclusions

This article presented the approach of implementing a modular patient unit of the ECMO modular simulator. The patient unit is responsible for most of the simulation scenarios that can be fully controlled and monitored using the instructor tablet. The patient unit allows to add, modify, remove, and maintain simulation modules without the need to upgrade or change the patient unit case or the main control board. This allows the instructor to add new simulation scenarios throughout training to offer training for multiple serious and even rare cases.

The next developmental step would be to create a mesh network between all the simulation networks in order for the main control board to control all of the simulation modules, even if they are far from the patient unit. This will improve the modularity of the whole system and for all units. Such a network will allow the instructor to add new simulation units without the need to have a physical control wire between the main control board and the simulation module. 

From a clinical point of view, a comprehensive evaluation study that utilizes the modular patient unit in collaboration with HMC is planned. The study will allow to enhance the user experience of the patient unit and determine how durable the control board and the case are after multiple uses during training sessions. The feedback will be taken into consideration to enhance the patient unit even more and, hence, implement it at a product level to redefine ECMO SBT standards worldwide.

## 7. Patents

The following US/PCT patent application has been filed: A. Alsalemi et al., “Using thermochromic ink for blood simulation in medical training,” US20190251869A1, 15 August 2019 and A. Alsalemi et al., “Using thermochromic ink for blood simulation in medical training,” WO2019159051A2, 22 August 2019.

## Figures and Tables

**Table 1:** Review of related work in ECMO SBT.

Work	Level of Fidelity	Advantages	Limitations
NIJMEGEN ECMO Simulator [21]	High	ECMO parameter simulation Can be controlled wirelessly Packaging portable Configurable system	Requires an external ECMO machine Lacks pre-programmed simulation scenarios Lacks color change simulation and line chattering
CLR [25]	Medium	Screen shows ECMO patient parameters Can be controlled wirelessly Cannulation simulation	Requires an external ECMO machine Lacks pre-programmed simulation scenarios Limited parameter control
Orpheus Perfusion Simulator [20]	High	Normal and emergency scenario simulation	Requires ECMO machine Does not use real blood
ECMO Patient Simulator (EPS) [26]	High	ECMO and patient parameter simulation Customizable scenarios	Uses actual blood Requires an external ECMO machine
3Dmed ECMO Simulation Kit [19]	Low	Employs artificial blood Cannulation simulation Simulates machine connection	Requires an external ECMO machine Limited simulation scenarios Lacks control interface
ECMO Mannequin by Puślecki et al. [22,23]	Medium	Includes a hydraulic system for pressure change simulation Cannulation simulation Cost-effective	Requires an external ECMO machine Lacks wireless control interface
Modular ECMO Simulator (this work, [29]	High	Modular design Blood simulation and related scenarios Cost-effective	Simulated blood fluid needs to be replaced every 12 h

## Data Availability

Not applicable.
